# Angiotensin II Exposure In Vitro Reduces High Salt-Induced Reactive Oxygen Species Production and Modulates Cell Adhesion Molecules’ Expression in Human Aortic Endothelial Cell Line

**DOI:** 10.3390/biomedicines12122741

**Published:** 2024-11-29

**Authors:** Nikolina Kolobarić, Nataša Kozina, Zrinka Mihaljević, Ines Drenjančević

**Affiliations:** Department of Physiology and Immunology, Faculty of Medicine Osijek, J. J. Strossmayer University of Osijek, J. Huttlera 4, 31000 Osijek, Croatia; nbdujmusic@mefos.hr (N.K.); nkozina@mefos.hr (N.K.); zmihaljevic@mefos.hr (Z.M.)

**Keywords:** angiotensin II (AngII), cell adhesion molecules (CAMs), endothelium, reactive oxygen species (ROS), sodium chloride (NaCl)

## Abstract

**Background/Objectives**: Increased sodium chloride (NaCl) intake led to leukocyte activation and impaired vasodilatation via increased oxidative stress in human/animal models. Interestingly, subpressor doses of angiotensin II (AngII) restored endothelium-dependent vascular reactivity, which was impaired in a high-salt (HS) diet in animal models. Therefore, the present study aimed to assess the effects of AngII exposure following high salt (HS) loading on endothelial cells’ (ECs’) viability, activation, and reactive oxygen species (ROS) production. **Methods**: The fifth passage of human aortic endothelial cells (HAECs) was cultured for 24, 48, and 72 h with NaCl, namely, the control (270 mOsmol/kg), HS320 (320 mOsmol/kg), and HS350 (350 mOsmol/kg). AngII was administered at the half-time of the NaCl incubation (10^−4^–10^−7^ mol/L). **Results**: The cell viability was significantly reduced after 24 h in the HS350 group and in all groups after longer incubation. AngII partly preserved the viability in the HAECs with shorter exposure and lower concentrations of NaCl. Intracellular hydrogen peroxide (H_2_O_2_) and peroxynitrite (ONOO^−^) significantly increased in the HS320 group following AngII exposure compared to the control, while it decreased in the HS350 group compared to the HS control. A significant decrease in superoxide anion (O_2_^.−^) formation was observed following AngII exposure at 10^−5^, 10^−6^, and 10^−7^ mol/L for both HS groups. There was a significant decrease in intracellular adhesion molecule 1 (ICAM-1) and endoglin expression in both groups following treatment with 10^−4^ and 10^−5^ mol/L of AngII. **Conclusions**: The results demonstrated that AngII significantly reduced ROS production at HS350 concentrations and modulated the viability, proliferation, and activation states in ECs.

## 1. Introduction

Complex interactions between vascular smooth muscle cells (VSMCs), endothelial cells (ECs), and immune cells contribute to vascular health and its physiological function [1,2]. Endothelial dysfunction (ED), an early indicator of vascular dysfunction (VD), is characterized by the loss of the endothelium’s anti-inflammatory, anticoagulant, and vasodilatory properties, marking one of the initial stages in cardiovascular disease (CVDs) [3,4,5,6], such as hypertension, atherosclerosis, stroke, obesity, diabetes, and thrombosis [7,8,9,10,11].

A powerful stimulator of vascular inflammation and oxidative stress is a high intake of sodium chloride (NaCl) [12,13,14], which leads to ED by promoting vascular low-grade inflammation and impaired vasodilation even in healthy, normotensive individuals [13,14,15,16]. For example, in young, healthy individuals, a seven-day high salt (HS) intake significantly impairs endothelium-dependent vasodilation in macro- and microcirculation without affecting arterial blood pressure (BP), fluid retention, or body composition [17]. The HS diet is known to increase the risk of heart failure and hypertension [18,19,20,21,22]. Excessive salt intake is a significant, yet preventable, contributor to CVD-related deaths globally. Implementing appropriate dietary restrictions, lifestyle modifications, and effective pharmacological interventions, all of which emphasize sodium reduction to help regulate blood pressure, improve endothelial function [23], and mitigate these negative trends [19,24,25,26,27,28]. Pharmacological treatments for hypertension include antihypertensive medications, such as angiotensin-converting enzyme (ACE) inhibitors [29,30,31], angiotensin II (AngII) receptor blockers (ARBs) [32,33,34,35,36], calcium channel blockers (CCBs) [37,38,39], and diuretics.

The effects of an HS diet are associated with alterations in the redox equilibrium between reactive oxygen species (ROS) generation and antioxidant mechanisms [40]. The bioavailability and production of endothelium-derived vasodilator nitric oxide (NO), along with other endothelium-derived vasodilator factors, are heavily influenced by increased ROS and overall vascular oxidative stress [13,41,42].

Notably, HS intake suppresses the renin–angiotensin system and AngII levels, which is the main regulatory system for the maintenance of blood volume and arterial blood pressure by vasoconstriction, increased aldosterone synthesis, and stimulation of the sympathetic nervous system [43,44,45]. In the vasculature, under normal conditions, higher levels of AngII induce ROS generation in vascular smooth muscle and ECs [46]. However, paradoxically, low levels of AngII also lead to increased ROS generation. For example, Ćosić et al. (2016) reported a significant increase in intracellular ROS levels and down-regulation of antioxidant enzyme expression, accompanied by impaired flow-induced dilation (FID) of middle cerebral arteries (MCAs) in Sprague-Dawley (SD) rats after a seven-day HS dietary intake [42]. On the other hand, subpressor low-dose exposure to AngII has shown beneficial effects on redox balance and restoration of flow-induced dilation after salt-induced impairment of endothelium-dependent vasodilation [47,48]. Interestingly, we have demonstrated that increased NaCl dietary intake induced low-grade systemic inflammation, which could be related to suppressed AngII levels and involved changes in Th17 and Treg cell distribution, a shift in lipid and arachidonic acid metabolism, and vascular wall remodeling in animal models [49] and in humans on an HS diet [13]. Since HS interferes with cellular response through the alteration of key molecules involved in the inflammatory response [13,50,51], using supraphysiological doses of sodium in vitro helps to model pathological conditions, amplify cellular responses, and reveal mechanisms that are not observable under normal baseline conditions. Specifically, it was shown that excess salt leads to cellular senescence, promoting inflammatory/fibrotic response and reducing NO generation in human and animal cells [52,53,54]. A previous study by Dmitrieva and Burg (2015) [55] showed that elevated extracellular NaCl affects adhesion molecules’ gene expression in human umbilical vein endothelial cells (HUVECs), directly involving it in the facilitation of vascular changes. Nevertheless, existing research does not appear to address the effects of AngII supplementation in a cell culture model of high NaCl intake, nor its implications for regulating redox balance in ECs. Thus, the present study hypothesizes that increasing concentrations of NaCl in the cell medium will affect the viability and ROS generation in ECs and will alter the activation state of the cells. At the same time, AngII addition in physiological doses will prevent these changes and exhibit a protective effect in the EC culture model.

The aim of this study was to assess the NaCl-dose response accompanied by AngII exposure in vitro on human aortic endothelial cells (HAECs) viability, intracellular ROS production, and cell adhesion molecules’ (CAMs’) expression (intercellular adhesion molecule 1, ICAM-1; vascular cell adhesion molecule 1, VCAM-1; E-selectin; and endoglin) following treatment, to further elucidate underlying mechanisms and effectors in salt-induced endothelial damage.

## 2. Materials and Methods

### 2.1. Materials and Chemical Reagents

HAECs were purchased from Innoprot (Barcelona, Spain). Cell culture flasks and well plates were purchased from TPP Techno Plastic Products AG (Trasagiden, Switzerland). Human Large Vessel Endothelial Cell Basal Medium, low serum growth supplement (LSGS), and Trypsin-EDTA (0.25%) were purchased from Gibco (Thermo Fisher Scientific, Waltham, MA, USA). AngII was purchased from Merck (Darmstadt, Germany). NaCl was purchased from Gram-Mol d.o.o. (Zagreb, Croatia). The 3-(4, 5-dimethylthiazolyl-2)-2 and 5-diphenyltetrazolium bromide (MTT) were purchased from Invitrogen (Thermo Fisher Scientific, Waltham, MA, USA).

### 2.2. HAECs

The HAECs were carefully thawed, seeded in tissue-appropriate cell culture flasks for adherent cells (T-25 cm^2^), and placed in an incubator (Shel Lab, CO_2_ Series, Sheldon Manufacturing Inc., Cornelius, OR, USA) under the following conditions: ~37 °C, 5% CO_2_, and >80% humidity level. Human Large Vessel Endothelial Cell Basal Medium supplemented with LSGS was used throughout our experiment. HAECs were monitored daily, and basal media was changed every other day until reaching confluence. For experimental purposes, the fifth passage (P5) of cells was used at approximately 80% confluence. Trypsinization was used as a method for the detachment of the HAECs from the flask/plate surface.

### 2.3. Cell Culture Treatment: NaCl and AngII

Cell culture treatment was performed in 24 and 96-well plates, depending on the following protocols. The osmolality of the control medium was 270 mOsmol/kg (133 mmol/L) (CTRL group). The HS medium was prepared by adding NaCl to the total osmolality of (1) 320 mOsmol/kg (158 mmol/L; HS320 group) and (2) 350 mOsmol/kg (173 mmol/L; HS350 group) [55,56,57,58]. During the experiment, the control medium was replaced by the high NaCl medium for 24, 48, and 72 h. The cell medium used for experimental purposes was not supplemented with LSGS, or in other words, it was serum-free.

Different concentrations of AngII were administered at half of the NaCl incubation (for 24 h after 12 h, for 48 h after 24 h, and for 73 h after 36 h). AngII was added in the following concentrations: 10^−4^, 10^−5^, 10^−6^, and 10^−7^ mol/L.

For experiments including only HS exposure and flow cytometry analysis of CAMs’ expression, 5 biological replicates (n = 5) were performed, with each replicate carried out over a 6-month period, using newly thawed vials of cells and conducted at the appropriate passage. However, for oxidative stress analysis, which included AngII exposure, 3 biological replicates (n = 3) were performed due to technical challenges encountered during the study period. All experiments were performed in 5 technical replicates.

### 2.4. Cell Viability and Metabolic Activity

MTT assay was performed as described by Shiwakoti et al. (2020) [59]. Cells were seeded in 96-well plates and treated with appropriate NaCl concentrations and 10^−6^ mol/L of AngII, as described above. After 24, 48, and 72 h, 10 µL of MTT stock solution was added to each well, and the plate was placed in an incubator for 4 h to produce formazan crystals. After incubation, 100 µL of MTT solvent was added to dissolve the created formazan crystals. The intensity of staining was proportional to the number of living cells. Plates were read spectrophotometrically at 595 nm using a microplate reader (BioRad PR 3100 TSC, Bio-Rad Laboratories, Hercules, CA, USA).

Cell viability was calculated as a percentage of the untreated control by subtracting the absorbance of treated cells from the absorbance of untreated cells, dividing by the absorbance of untreated cells, and multiplying by 100.

### 2.5. Flow Cytometry

The samples were collected after the HAECs were exposed for 72 h to different concentrations of NaCl and AngII (36 h), which were administered to the cell cultures in 24-well plates. The cells were washed in 1× phosphate-buffered saline (PBS) and prepared for the appropriate staining protocol (approximately 10^5^ cells/mL of medium).

The FACS Canto II flow cytometer (BD Bioscience, Franklin Lakes, NJ, USA; 488 excitation laser and 530/30 BP analysis filter) was used for the assessment of intracellular ROS production, as well as the CAMs’ expression. Data analysis and visualization were performed using Flow Logic software v.8 (Inivai Technologies, Mentone, Australia).

#### 2.5.1. Intracellular ROS Production

A dichlorofluorescein diacetate (DCF-DA) assay was used to determine the levels of hydrogen peroxide (H_2_O_2_) and peroxynitrite (ONOO^−^). DCF-DA is a non-fluorescent, cell-permeable dye that is deacetylated inside the cell by cellular esterases to DCFH (2′,7′-dichlorodihydrofluorescein). DCFH is oxidized in the presence of ROS in a fluorescent DCF (2′,7′-dichlorofluorescein), which can be detected as an indicator of oxidative stress. The dihydroethidium (DHE) assay was used to determine the level of superoxide anion (O_2_^.−^) in the HAECs. This is another cell-permeable dye, which, upon entering the cell, is oxidized by superoxide to form ethidium, which emits red fluorescence that can be quantified to assess the levels of superoxide production within the cell.

Cells were resuspended in 100 µL of 1× PBS and incubated with DCF-DA or DHE (10 µM final concentration) for 30 min at +4 °C. Following the incubation period, the samples stained with DCF-DA were re-suspended in PBS and immediately analyzed, while samples stained with DHE required additional rinsing with PBS before resuspension in PBS and cytometer reading. Following initial readings, phorbol 12-myristate 13-acetate (PMA) was added to each sample to stimulate ROS production. Data are expressed as geomean fluorescence intensity (GMFI) in FLH-1 and FLH-2 channels. Assays were performed according to previously reported protocols that were optimized for this purpose [60,61].

#### 2.5.2. CAMs’ Expression

Cells were resuspended and washed twice before staining with appropriate antibodies. The following antibodies were used for staining the HAECs: ICAM-1 (CD54, clone: 15.2, Proteintech Group, Inc., Rosemont, IL, USA), VCAM-1 (CD106, clone: 51-10C9; BD Pharmigen Inc., Franklin Lakes, NJ, USA), E-selectin (CD62E, clone: 68-5H11; BD Pharmigen Inc., Franklin Lakes, NJ, USA), and endoglin (CD105, clone: 266; BD Pharmigen Inc., Franklin Lakes, NJ, USA).

### 2.6. Statistical Analysis

The normality of the residuals, where appropriate, was assessed using the Shapiro–Wilk test, while the homogeneity of variances was determined using the Brown–Forsythe test. Significant differences between treatment groups were determined using one-way ANOVA and the following post hoc analyses: Dunnett’s test, which compared each treatment group to the control, and Tukey’s HSD test, which compared all treatment groups against each other (*p* < 0.05).

## 3. Results

### 3.1. Assessment of Cell Metabolic Activity (MTT Assay)

**Figure 1:**
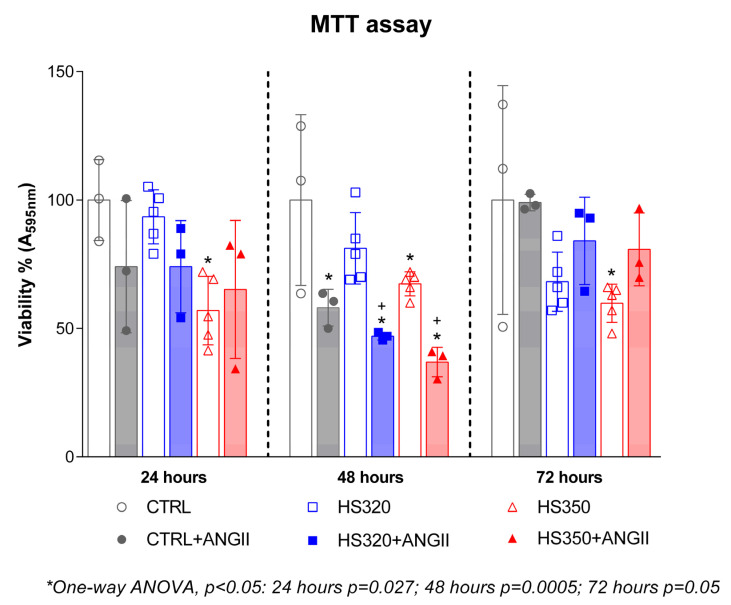
Cellular metabolic activity of HAECs after treatment with different concentrations of NaCl and AngII assessed via MTT assay. A—absorbance; nm—nanometers; CTRL—control; HS—high salt; AngII—angiotensin II; One-way ANOVA; *^,+^ significance level *p* < 0.05 (* compared to control group; ^+^ compared to HS group before AngII).

Changes in cellular metabolic activity (i.e., an indicator of cell viability) of HAECs following incubation with different NaCl concentrations before and after the addition of AngII in the cell media are shown in Figure 1. Cell metabolic activity was significantly reduced after 24, 48, and 72 h at 350 mOsmol/kg NaCl concentration compared to the CTRL. The addition of AngII to the HS320 incubated cells did not alter cell metabolic activity at 24 and 72 h time points, except for 48 h, where AngII significantly decreased metabolic activity compared to the CTRL and HS320 conditions. The addition of AngII to the HS350 incubated cells resulted in preserved metabolic activity of cells after 24 and 72 h, while it significantly decreased metabolic activity after 48 h of incubation.

### 3.2. Assessment of Intracellular ROS Production: Analysis of DCF-DA and DHE Fluorescence Signals

**Figure 2:**
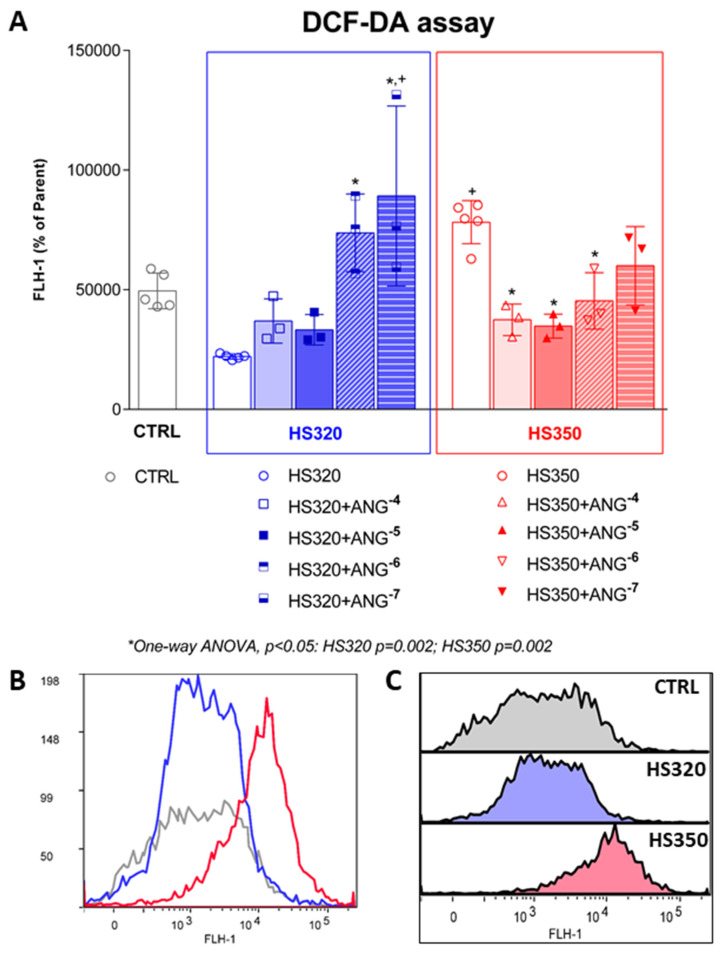
Formation of hydrogen peroxide and peroxynitrite in HAECs following high-salt treatment accompanied by AngII exposure (**A**). Representative histogram overlay (**B**) and stacked histograms (**C**). Grey color representing CTRL group, blue color representing HS320 group, red color representing HS350 group. Results are expressed as geometric mean fluorescence intensity (GMFI). DCF-DA—dichlorofluorescein diacetate; CTRL—control; HS—high salt; One-way ANOVA; *^,+^ significance level *p* < 0.05 (* compared to HS group before AngII; ^+^ compared to control group).

In the HS320 group, treatment with NaCl did not have any significant effect on the formation of H_2_O_2_ and ONOO^−^ in the HAECs. Following 10^−4^ and 10^−5^ mol/L of AngII exposure, H_2_O_2_ and ONOO^−^ formation was not different from the levels observed in the HS320 condition without AngII but increased significantly with AngII 10^−6^ and 10^−7^ mol/L, suggesting that higher doses of AngII suppressed H_2_O_2_ and ONOO^−^ production. Formation of H_2_O_2_ and ONOO^−^ was significantly increased in the HS350 group compared to the CTRL group (Figure 2). In the HS350 group, a significant decrease was detected after administering AngII in concentrations from 10^−4^ to 10^−6^ mol/L.

**Figure 3:**
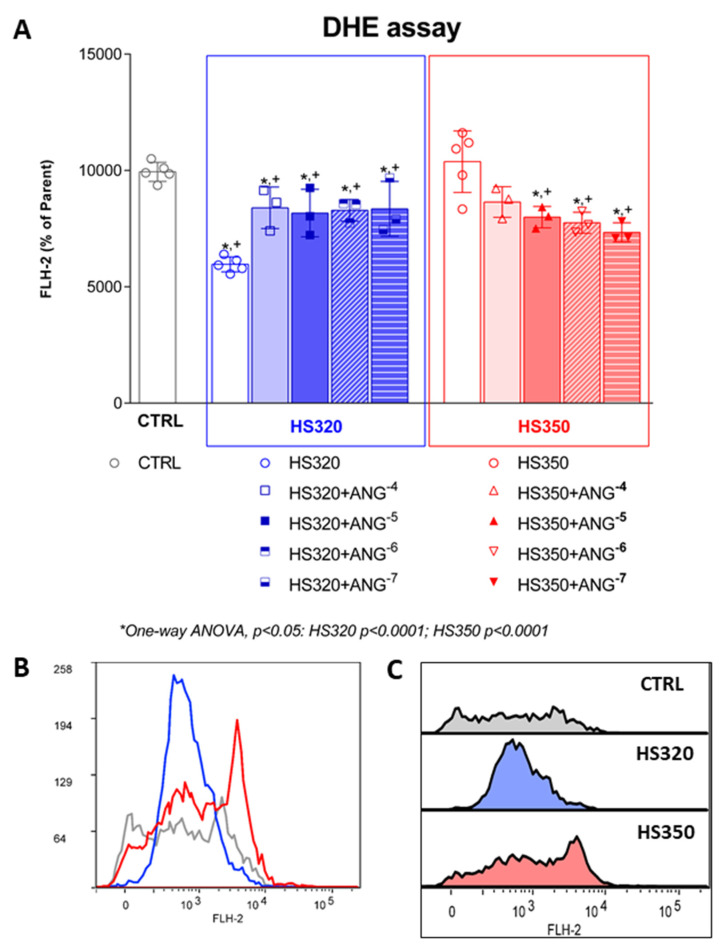
Formation of superoxide in HAECs following high-salt treatment accompanied by AngII exposure (**A**). Representative histogram overlay (**B**) and stacked histograms (**C**). Grey color representing CTRL group, blue color representing HS320 group, red color representing HS350 group. Results are expressed as geometric mean fluorescence intensity (GMFI). DHE—dihydroethidium; CTRL—control; HS—high salt; One-way ANOVA; *^,+^ significance level *p* < 0.05 (* compared to HS group before AngII; ^+^ compared to control group).

Production of O_2_^.−^ in HAECs was significantly decreased in HS320 compared to CTRL, while there were no significant changes in the formation of O_2_^.−^ in the HS350 group compared to the CTRL group (Figure 3). O_2_^.−^ level was significantly decreased in the HS320 group supplemented with AngII compared to the CTRL but increased compared to the HS320 group without AngII. In the HS350 group, O_2_^.−^ production was significantly decreased after the addition of AngII compared to the CTRL and HS350 group without AngII.

Additionally, PMA stimulation that was performed following the initial baseline readings, specifically under HS conditions (HS320 and HS350), is presented as Supplementary Figures for DCF-DA (Figure S1) and DHE assays (Figure S2).

### 3.3. Quantitative Analysis of CAM Expression: Flow Cytometry Analysis

**Figure 4:**
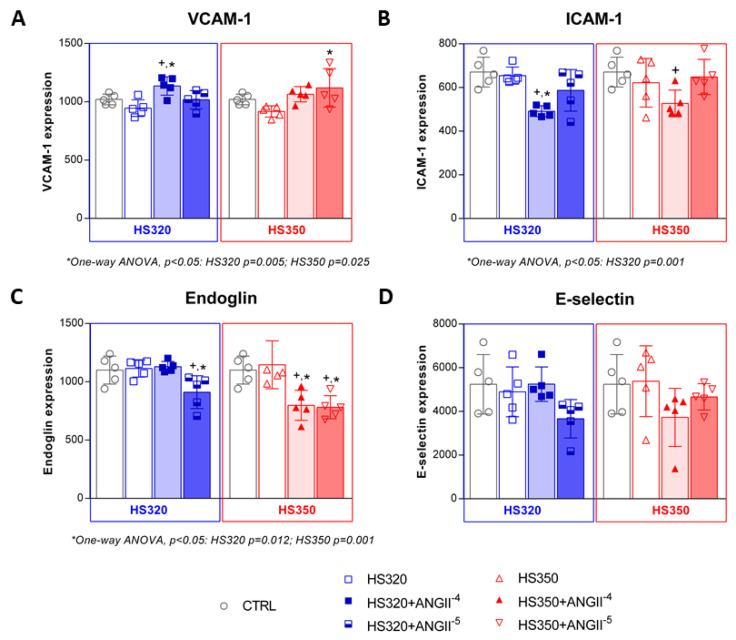
Changes in CAMs’ expression: VCAM-1 (**A**), ICAM-1 (**B**), Endoglin (**C**), and E-selectin (**D**) in HAECs following high-salt treatment accompanied by AngII exposure. Grey color representing CTRL group, blue color representing HS320 group, red color represent-ing HS350 group. Results are expressed as geometric mean fluorescence intensity (GMFI). CAMs—cell adhesion molecules; HAECs—human aortic endothelial cells; VCAM-1—vascular cell adhesion molecule 1; ICAM-1—intracellular adhesion molecule 1; HS—high salt; CTRL—control; AngII—angiotensin II; One-way ANOVA; *^,+^ significance level *p* < 0.05 (* compared to HS group before AngII; ^+^ compared to control group).

Changes in CAM expressions following treatment with NaCl and different concentrations of AngII are shown in Figure 4. HS did not affect VCAM-1 expression (Figure 4A). However, VCAM-1 was significantly increased in the HS320 group following the addition of AngII at a 10^−4^ mol/L dose compared to the CTRL and HS groups, and in the HS350 group following AngII at 10^−5^ mol/L compared to the HS group. ICAM-1 expression was significantly decreased in the HS320 and HS350 groups following the addition of 10^−4^ mol/L of AngII compared to the CTRL (Figure 4B).

A significant decrease in endoglin expression occurred in both HS groups supplemented with 10^−5^ mol/L of AngII compared to the HS and CTRL groups (Figure 4C). There were no significant changes in the expression of E-selectin in all study groups, with or without AngII exposure (Figure 4D).

### 3.4. Protein Expression of AT1 and AT2 Receptors

Results that are presented in the Supplementary Materials (Figure S3) showed that protein expression of both AT receptors was significantly decreased in animals on the HS diet compared to control animals. AngII partially restored the AT1 receptor expression compared to the HS group but was still lower than in the CTRL group. The AT2 receptor was not affected by the AngII treatment.

## 4. Discussion

Other than regulating vascular tone, blood flow, and coagulation, ECs also secrete various cytokines, chemokines, growth factors, and adhesion molecules. This makes them important players in inflammation and leukocyte-EC adhesion, which underlies all major cardio-metabolic diseases [4,7,62,63].

Some of the noteworthy findings in the present study are the following: (1) higher doses and prolonged exposure to NaCl decrease the ECs’ metabolic activity and increase the ECs’ ROS production, thus making ECs an important source of ROS leading to endothelial dysfunction; (2) AngII exposure significantly reduces ROS production of ECs and can protect their metabolic activity to some extent; (3) AngII can modulate the expression of CAMs (as demonstrated by a decrease in ICAM-1 and endoglin expression with certain utilized doses of AngII). Taken together, the present study demonstrates the direct effects of NaCl on ECs’ activation status and redox balance, which could be altered by AngII.

As previously discussed, HS intake is a significant source of ROS, leading to systemic oxidative stress, decreased antioxidant activity, and impaired endothelial function [64,65]. NaCl, being the universal stressor, provokes an adaptive response in cells to mitigate the damage and maintain viability under hyperosmotic conditions. Generally, the elevation of osmolality above 400 mOsmol/kg H_2_O progressively impairs cell proliferation, and while cell cycle arrest provides short-term adaptation, it ultimately leads to cell death (500 mOsmol/kg), while lower concentration for shorter periods induces reversible changes in cells [66]. In our current study, we aimed to evaluate the NaCl-dose and time-dependent response in conjunction with AngII exposure in vitro, focusing on the HAECs’ metabolic activity, intracellular ROS production, and the expression of CAMs (ICAM-1, VCAM-1, E-selectin, and endoglin) following treatment. The results suggest that a NaCl concentration of 320 mOsmol/kg in the HAECs culture has a hormetic effect (bi-phasic dose–response curve), yielding a lower level of ROS, especially superoxide anion [67,68,69,70]. There are several potential explanations for this occurrence. HS alters the redox state of cells. A low dose of this stressor might produce mild stress-altering gene expression and protein synthesis. As demonstrated earlier, this further affects enzymes involved in ROS production, such as NADPH oxidase and mitochondrial respiratory complex, activating protective mechanisms like antioxidant pathways and consequently leading to reduced ROS [64,66,71,72], suggesting that cellular tolerance occurs when cells are exposed to NaCl concentrations close to physiological levels, such as 320 mOsmol/kg. However, Dmitrieva et al. (2004) and others warned that these surviving, adapted cells may be damaged beyond repair at the DNA level despite rapid proliferation and minimal apoptosis [66,72,73,74]. Furthermore, Xu et al. (2009) co-cultured ECs with VSMCs and reported that ECs upregulate antioxidants, more accurately, thioredoxin, consequently decreasing ROS production by the VSMCs in a state of stress [75]. These findings are supportive of our present results but also present a challenging perspective for future investigations.

On the other hand, a concentration of 350 mOsmol/kg appears to be a critical point for NaCl-induced oxidative stress in our cultured HAECs, significantly increasing H_2_O_2_ and ONOO^−^ production (DCF-DA assay). A significant increase in O_2_^.−^ was observed at a NaCl concentration of 350 mOsmol/kg following PMA stimulation (DHE assay), presented in Figure S1 (Supplementary Materials). These differences in ROS production at different NaCl concentrations might result from different metabolic pathways activated by NaCl, ranging from oxidase activation to structural/mitochondrial damage [76,77,78].

The endothelium, a metabolically active layer of cells, acts as a selective barrier separating the vascular wall and circulating blood [79]. Its intricate role includes functioning as an active endocrine, paracrine, and autocrine organ necessary for maintaining vascular homeostasis [80,81,82]. It is well documented that higher supraphysiological concentrations of AngII increase ROS production via NADPH-oxidase [83,84]. Even at low concentrations, such as 10*−7* mol/L, AngII induces protein nitration in endothelial cells, with the extent of nitration increasing in a concentration-dependent manner as the concentration of AngII rises [85]. However, too-low levels of AngII have been shown to increase ROS in the vasculature, too [48,86,87], rendering the conclusion that a physiological range of AngII is needed to balance oxidative stress. Previously, we have shown that three days of a subpressor dose of AngII supplementation in HS-fed rats restored the FID of MCAs and significantly increased GPx4 and extracellular SOD antioxidative enzyme expression [48]. Furthermore, the HS diet significantly reduced the expression of Cu/Zn SOD and Mn SOD in the cerebral resistance arteries of the HS-fed rats, while an infusion of AngII restored protein Cu/Zn SOD (but not Mn SOD) expression [88]. A study involving the two-week infusion of AngII in sham rats and rats with myocardial infarction suggested the activation of counter-regulatory mechanisms by AngII, as blood pressure changes and vascular remodeling were minimal in the animals with myocardial infarction leading to compensation of hypertensive and growth stimulatory effects of AngII observed in sham rats. Namely, rats with myocardial infarction exhibited concomitant increases in plasma ANP and NO synthase activity following AngII infusion, demonstrating AngII-activated protective mechanisms related to blood vessel structure and function [89]. In the present study, AngII exposure in our cell cultures significantly altered metabolic activity following HS treatment after 48 h. Viability was reduced after 48 h but seemingly restored after 72 h of incubation. It has been reported that AngII exposure increases the viability of human mammary epithelial cells (184A1) through mitochondrial metabolism, as well as changes in cell behavior via AT1R overexpression [90]. Interestingly, AngII stimulates the Na/K pump in the proximal tubular cell culture and acutely stimulates the transcellular sodium transport [91], thus preventing an increase in cellular osmolality. If such an effect of AngII is present in the ECs, it remains to be investigated and demonstrates that it could also contribute a piece of the puzzle to the beneficial effect of AngII in the endothelium.

One possible explanation for an observed beneficial effect of AngII on the metabolic activity of the NaCl-stressed HAECs is the upregulation of antioxidative systems. This has been demonstrated in animal models of rats on an HS diet and in Dahl salt-sensitive rats [46,48]. In the vasculature, subpressor doses of AngII have led to restored microvascular reactivity and increased expression of antioxidative enzymes, such as SOD. Interestingly, it was previously reported that AngII, as a stress inducer, increases the expression of Nrf2-related genes in rat and mice neuronal cell lines, consequently leading to an increase in cell viability [92]. Consequently, future research should not only focus on the functions and activation pathways of Nrf2 but also explore its broader implications and applications.

The results showed that elevated levels of NaCl lead to an increase in oxidative stress and cause changes in the expression of certain CAMs. However, these changes do not occur uniformly in the same direction or at the same concentration of NaCl. This variability is likely due to the involvement of different signaling pathways, which may respond differently to NaCl, resulting in diverse effects on oxidative stress and the regulation of CAMs [13,93]. Endothelial pro-inflammatory phenotype is generated by increased pro-inflammatory cytokines production (IL-1β, IL-6, TNF-α) alongside C-reactive protein (CRP). This phenotype is characterized by an increase in CAMs, such as E-selectin, ICAM-1, and VCAM-1 [94,95,96]. Animal studies have shown upregulation in CAM expression following the HS diet [97,98,99]. Leukocyte migration and vascular adhesion mediated by ICAM-1 and VCAM-1 regulate inflammation and homeostasis in various diseases [98,100]. HS intake also stimulates the expression of E-selectin, monocyte chemotactic protein-1 (MCP-1), and endothelin 1 (ET1) [101,102]. ICAM-1 plays an important role in vascular inflammation and represents an attractive target for future treatment due to its increased expression in response to stressors [103,104]. The results of the present study showed decreased ICAM-1 expression following AngII exposure in a moderate NaCl concentration. Furthermore, endoglin is an angiogenesis/vascular remodeling participator with its soluble form that tends to be increased in inflammatory/vascular pathological conditions (atherosclerosis, hypertension, type 2 diabetes, and dyslipidaemia) and endothelial injury events [105,106,107]. In our study, endoglin expression was significantly decreased following AngII exposure in all three HS groups. This is in line with the notion that oxidative stress upregulated endoglin, e.g., in the placenta [108]. Since AngII can decrease oxidative stress, as demonstrated in this and previous studies, the reduction in endoglin levels is warranted.

While our study lays a foundation for future research on the mechanisms of AngII in relation to cell viability and activation, it has certain limitations. Subsequent studies should incorporate pharmacological experiments and explore the effects of angiotensin receptor blockers on ROS production and the expression of adhesion molecules in HAECs following AngII treatment. Indeed, in our previous work on rat animal models, we have demonstrated that the inhibition of AT1R leads to impaired endothelium-mediated vasodilation in response to changes in flow in isolated, pressurized, middle cerebral arteries and increases oxidative stress [93]. Further, results on protein expression of the AT1 and AT2 receptors in brain blood vessels (BBV) provided in Supplementary Figure (Figure S3) suggest that permissive effects of AngII on vasodilation and oxidative balance include and are mediated mainly via AT1 receptors.

One limitation of this study that may arise is the osmolarity control of the experiments that could have been performed by, e.g., mannitol or sorbitol. However, in this study, we opted not to perform this experiment due to growing evidence that increasing the osmolarity of the culture medium by the addition of sorbitol or mannitol (100 mM) did not show alteration in cellular response, as shown in neutrophils [56] or rat glial cell culture [109], but results suggested the sole effect of high sodium and not of increased osmolarity. Furthermore, the mechanisms of action and consequences of mannitol vs. NaCl are different [110], and mannitol also induces apoptosis in ECs [111]. Importantly, the effect of increased osmolarity is reversible [112], as shown in the experiments on mIMCD3 mouse renal collecting duct cells, where, with up to 600 mosmol/kg, the effect was only transient, and by 12 h at 550 mosmol/kg, the effect was reversible to normal. Thus, our control condition is the physiological concentration of NaCl (i.e., 270 mOsmol/L).

While our study focused on measuring intracellular ROS (baseline and following PMA stimulation), we acknowledge that calcium signaling, which can modulate PKC activity, was not directly assessed. This represents a limitation of our study, particularly in the context of HS conditions where calcium-dependent PKC-signaling pathways are relevant [113]. Future research should explore this aspect, including the measurement of intracellular calcium levels and their relationship to PKC activation and ROS generation.

## 5. Conclusions

The present study demonstrated that a longer duration and higher concentration of NaCl have detrimental effects on ECs’ metabolic activity and induce them as a source of ROS. Importantly, the present study demonstrated that AngII can decrease oxidative stress and alter the activation state of ECs, thus providing beneficial conditions for cell survival. The potential common denominator of observed effects of AngII is the upregulation of antioxidative systems. Thus, future research should focus on further elucidating the mechanisms underlying AngII-induced antioxidative defense modulation and its potential therapeutic implications.

## Data Availability

The original contributions presented in this study are included in the article/Supplementary Materials. Further inquiries can be directed to the corresponding author.

## Supplementary Materials

The following supporting information can be downloaded at: https://www.mdpi.com/article/10.3390/biomedicines12122741/s1, Figure S1: Intracellular production of hydrogen peroxide and peroxynitrite (DCF-DA) in HAECs following high-salt treatment without and upon stimulation with PMA. Results are expressed as geometric mean fluorescence intensity (GMFI). DCF-DA–dichlorofluorescein diacetate; HAECs–human aortic endothelial cells; CTRL–control; HS–high salt; PMA–phorbol 12-myristate 13-acetate; One-way ANOVA; * significance level *p* < 0.05; Figure S2: Intracellular production of superoxide anion (DHE) in HAECs following high-salt treatment without and upon stimulation with PMA. Results are expressed as geometric mean fluorescence intensity (GMFI). DHE–dihydroethidium; HAECs–human aortic endothelial cells; CTRL–control; HS–high salt; PMA–phorbol 12-myristate 13-acetate; One-way ANOVA; * significance level *p* < 0.05; Figure S3: The relative protein expression (normalized to β-actin as a reference protein and loading control) and representative blots of the AT1 and AT2 receptors in brain surface vessels in the CTRL, HS, and HS and ANGII groups of Sprague-Dawley rats determined by Western blot method. CTRL–control; HS–high salt; ANGII–angiotensin II; AT1–angiotensin II type 1; AT2–angiotensin II type 2; One-way ANOVA; * significance level *p* < 0.05.

## Abbreviations

ACE	angiotensin-converting enzyme
AngII	angiotensin II
ANOVA	analysis of variance
ARBs	angiotensin II receptor blockers
AT1R	angiotensin type 1 receptor
CAMs	cellular adhesion molecules
CCBs	calcium channel blockers
CO_2_	carbon dioxide
CRP	C-reactive protein
Cu	copper
DCFDA	dichlorofluorescein diacetate
DCFH	2′,7′-dichlorofluorescein
DHE	dihydroethidium
ET-1	endothelin 1
FID	flow-induced dilation
FLH	fluorescence height
GMFI	geometric mean fluorescence intensity
GPx	glutathione peroxidase
H_2_O_2_	hydrogen peroxide
HAECs	human aortic endothelial cells
HS	high salt
HUVECs	human umbilical vein endothelial cells
ICAM-1	intercellular adhesion molecule 1
IL	interleukin
MCAs	middle cerebral arteries
MCP-1	monocyte chemotactic protein-1
Mn	manganese
MTT	3-(4, 5-dimethylthiazolyl-2)-2, 5-diphenyltetrazolium bromide
NaCl	sodium chloride
NADPH	nicotinamide adenine dinucleotide phosphate
Na/K	sodium/potassium pump
NO	nitric oxide
O_2_^.−^	superoxide anion
ONOO^−^	peroxynitrite
PBS	phosphate-buffered saline
PKC	protein kinase C
PMA	phorbol 12-myristate 13-acetate
Prx	peroxiredoxin
ROS	reactive oxygen species
SD	Sprague-Dawley
SOD	superoxide dismutase
Th17	T helper 17 cells
TNF-α	tumor necrosis factor alpha
Treg	T regulatory cells
VCAM-1	vascular adhesion molecule 1
Zn	zinc
