# Supplementary material for: Angiotensin II Exposure In Vitro Reduces High Salt-Induced Reactive Oxygen Species Production and Modulates Cell Adhesion Molecules’ Expression in Human Aortic Endothelial Cell Line
Source: Biomedicines. 2024 Nov 29;12(12):2741. doi: 10.3390/biomedicines12122741 (PMC11726729; doi:10.3390/biomedicines12122741)
Supplement: Supplementary file 1 [file biomedicines-12-02741-s001.zip › biomedicines-3330533-supplementary/Animal housing, sampling and WB.pdf]

Ethical approval

All experimental procedures conformed to the European Guidelines for the Care and Use of Laboratory Animals (directive 2010/63/EU). They were approved by the local ethical committee (Faculty of Medicine, University of Osijek; Class: 602-04/14-08/06; No.: 2158-61-07-14-119) and the competent authority, the Ministry of Agriculture, Croatia (HR-POK-005).

Animals

The rats were housed at the animal care facility (nationally registered and certified user/breeder of mice and rats for educational and scientific purposes) of the Faculty of Medicine at the Josip Juraj Strossmayer University of Osijek, Croatia. Animals were housed in standard plastic cages in a temperature and humidity-controlled environment, with a 12:12 h light–dark cycle, and were allowed access to food and water *ad libitum*. As many samples as possible were taken from the same animal to reduce the number of used animals.

Experimental groups

Healthy 10-week-old male Sprague-Dawley rats were randomly divided into three groups, with 5–10 animals per group depending on the specific experiment: a low-salt diet group (0.4% NaCl in chow; Mucedola, Italy), a high-salt diet group (4% NaCl in chow; Mucedola, Italy) for 1 week, and a high-salt diet with angiotensin II (HS+ANG II) group. In the HS+ANG II group, an osmotic minipump (ALZET, Cupertino, CA, USA) was implanted on day 4 to administer angiotensin II (100 ng/kg per min) subcutaneously for 3 days (days 4–7). Both the low-salt and high-salt groups underwent a sham procedure with pumps filled with physiological saline, matching the volume used to prepare angiotensin II for the HS+ANG II group.

Western blot

Protein levels of AT1 and AT2 receptors were measured in isolated cerebral blood vessels taken from the brain surface. Blood vessels from two rats within the same treatment group were combined to form each sample. Our laboratory's established protocol for protein extraction and homogenization, previously published, was used here. Tissues were pulverized in liquid nitrogen, then homogenized on ice in a buffer containing 10 mmol/L Tris base, 1 mmol/L EDTA, 0.4% SDS (Acros Organics), and a Protease Inhibitor Cocktail (4 mL/100 mL), followed by centrifugation at 17,000g for 30 minutes at 4°C. SDS-PAGE electrophoresis was carried out using Bio-Rad's Mini PROTEAN Tetra Cell and Criterion blotter (100V) with subsequent blotting (200 mA, 2 hours) onto PVDF membranes.

Chemiluminescence detection was performed following the instructions for Pierce ECL Western Blotting Substrate, with signals captured on a Bio-Rad ChemiDoc Imager (Bio-Rad Laboratories, CA, USA). Protein expression levels were analyzed using ImageJ software (National Institutes of Health) according to the developer's guidelines. Expression levels were normalized to β-actin and presented as relative protein levels. Detection of target proteins was done using rabbit antibodies from Abcam, UK: AT1R (ab-18801, 1:1000) and AT2R (ab-EPR3876, 1:1000), a mouse monoclonal β-actin antibody (Santa Cruz Biotechnology, sc-4778, 1:7500), a goat anti-mouse HRP-labeled secondary antibody (Santa Cruz Biotechnology, sc-2005, 1:10000), and a goat anti-rabbit HRP-labeled secondary antibody (Abcam, UK, ab-205718, 1:10000).
