# Supplementary material for: Angiotensin II Exposure In Vitro Reduces High Salt-Induced Reactive Oxygen Species Production and Modulates Cell Adhesion Molecules’ Expression in Human Aortic Endothelial Cell Line
Source: Biomedicines. 2024 Nov 29;12(12):2741. doi: 10.3390/biomedicines12122741 (PMC11726729; doi:10.3390/biomedicines12122741)
Supplement: Supplementary file 1 [file biomedicines-12-02741-s001.zip › biomedicines-3330533-supplementary/Supplemetary figures.pdf]

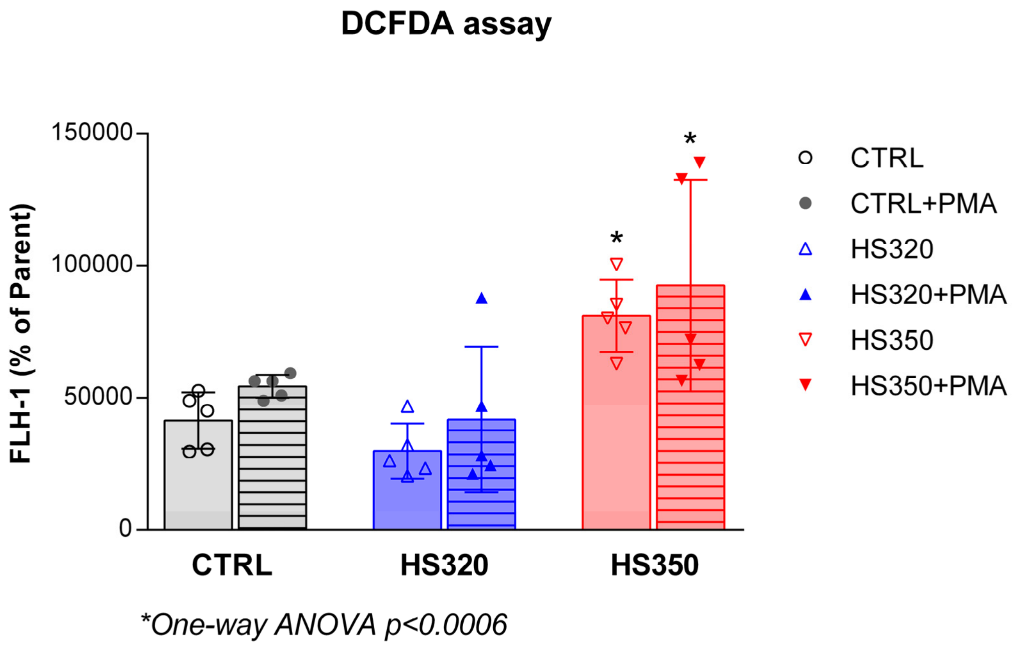

Figure S1. Intracellular production of hydrogen peroxide and peroxynitrite (DCF-DA) in HAECs following high-salt treatment without and upon stimulation with PMA. Results are expressed as geometric mean fluorescence intensity (GMFI). DCF-DA - dichlorofluorescein diacetate; HAECs – human aortic endothelial cells; CTRL – control; HS – high salt; PMA - phorbol 12-myristate 13-acetate. One-way ANOVA; * significance level *p* < 0.05.

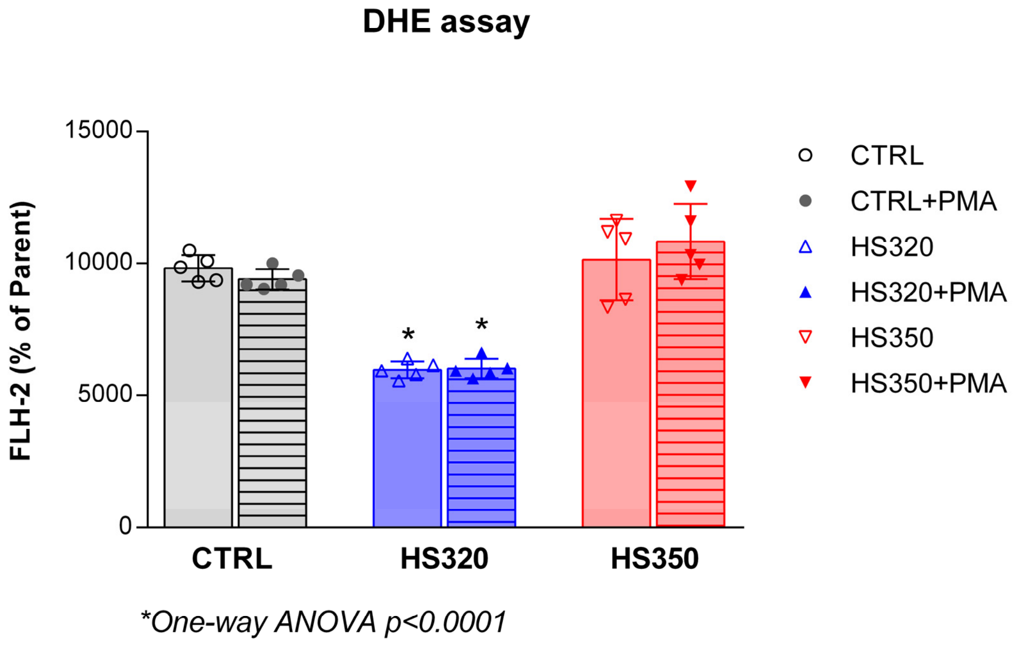

Figure S2. Intracellular production of superoxide anion (DHE) in HAECs following high-salt treatment without and upon stimulation with PMA. Results are expressed as geometric mean fluorescence intensity (GMFI). DHE – dihydroethidium; HAECs – human aortic endothelial cells; CTRL – control; HS – high salt; PMA - phorbol 12-myristate 13-acetate. One-way ANOVA; * significance level *p* < 0.05.

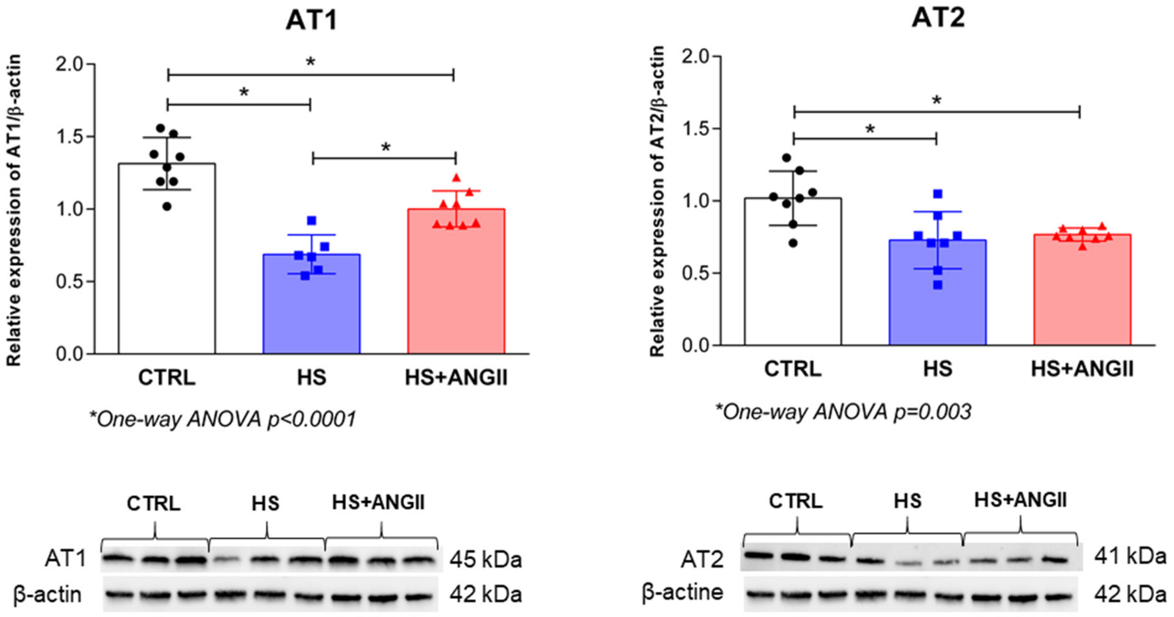

Figure S3. Relative protein expression (normalized to β-actin as a reference protein and loading control) and representative blots of AT1 and AT2 receptors in brain surface vessels in CTRL, HS and HS+ANGII groups of Sprague-Dawley rats determined by Western blot method. CTRL – control; HS – high salt; ANGII – angiotensin II; AT1 – angiotensin II type 1; AT2 – angiotensin II type 2. One-way ANOVA; * significance level $p < 0.05$.
