# Supplementary figures and images for: Angiotensin II Exposure In Vitro Reduces High Salt-Induced Reactive Oxygen Species Production and Modulates Cell Adhesion Molecules’ Expression in Human Aortic Endothelial Cell Line
Source: Biomedicines. 2024 Nov 29;12(12):2741. doi: 10.3390/biomedicines12122741 (PMC11726729; doi:10.3390/biomedicines12122741)

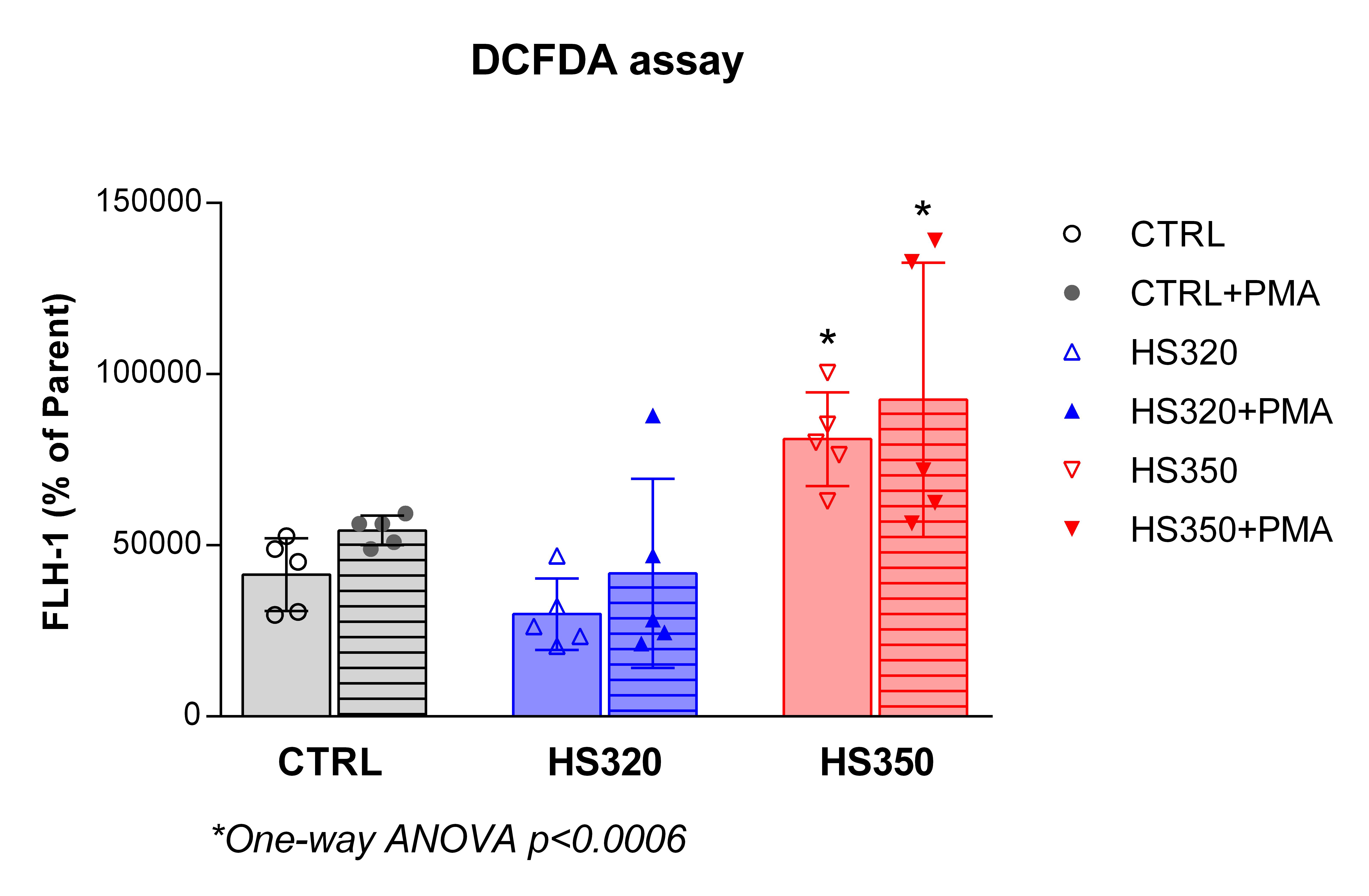

Supplement: Supplementary file 1 [file biomedicines-12-02741-s001.zip › biomedicines-3330533-supplementary/Figure S1. DCF-DA Assay.png]

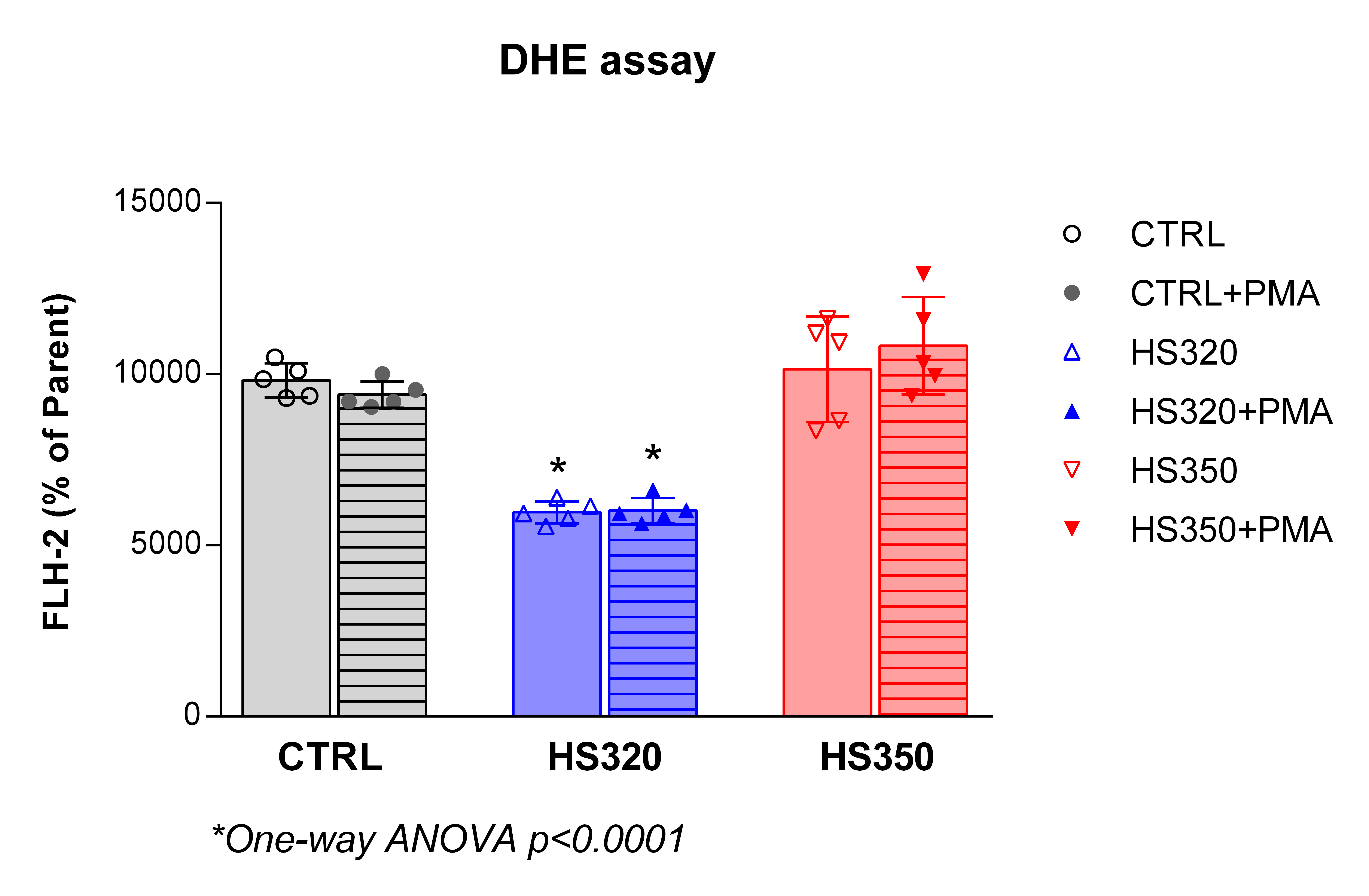

Supplement: Supplementary file 1 [file biomedicines-12-02741-s001.zip › biomedicines-3330533-supplementary/Figure S2. DHE Assay.png]

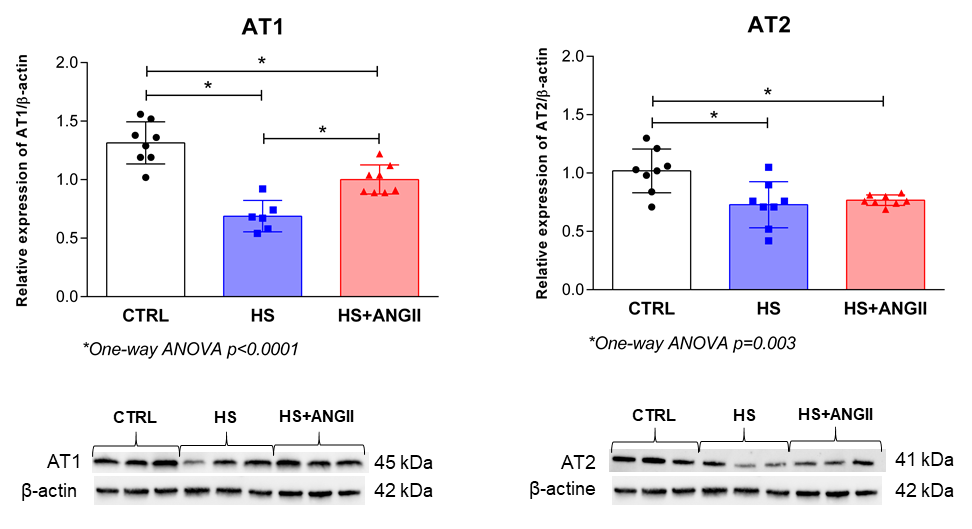

Supplement: Supplementary file 1 [file biomedicines-12-02741-s001.zip › biomedicines-3330533-supplementary/Figure S3. Western blot AT1 and AT2 receptors.png]
